# Research into the Action of Mercury, Podophylline, and Taraxacum on the Biliary Sectetion, Etc.

**Published:** 1870-03

**Authors:** 


### Research into the Action of Mercury, Podophylline, and Taraxacum on the Biliary Sectetion, etc.

[The following article is a view of Dr. Bennett's pamphlet on this subject, by one of the Editors of the Practitioner.]

An investigation of the action or want of action of mercury and taraxacum in influencing secretion of bile, is something like an inquiry by Dr. Colenso into the authenticity of the Book of Numbers. It is something to make the cheeks of many and many an old-fashioned practitioner turn pale, to shake the whole foundation of his therapeutical creed, to symbolise the end of all things at least as regards drugs. Podophylline is but a juvenile. That podophylline, albeit lauded in no measured terms for its flow-of-bile-producing qualities, should prove a traitor, was a blow which some little exertion of moral courage could enable the believer to survive; but that any doubt should be felt about the capacities of calomel and blue pill, and extractum taraxaci, in "acting upon the liver," must have conveyed a shock in various quarters, from which we should imagine there are many still suffering. The investigation, however, has taken place. It has been conducted by men of proved ability. Its results are here placed before the reader in very intelligible form, and we confess, for our part, that at these results we are not one whit surprised. The experiments clearly show, what careful observation in practice must have taught the unprejudiced, that faith in the cholagogue action of these drugs has been misplaced, that our trust in blue pill from this particular point of view has been as ill-judged as it was strong, and that the days are rapidly drawing to a close when a practitioner of medicine will be able, without subjecting himself to ridicule, to inform his patient that his complaint is "all liver," and requires sundry doses of mercury, podophylline, or taraxacum.

In the experiments adopted by the Committee, dogs were employed. The fundus of the gall-bladder was attached to the abdominal wall, and a fistulous opening made in it through which the whole of the bile, secreted for at least twenty-four hours at a time, was collected. During the five days on which mercury was given, the quantity of bile secreted was diminished to nearly a half of what it was in the period, the average amount of bile secreted was on the whole greater on the days when no mercury was given than on the other days.

Dr. Bennett comes to the conclusion that mercury, when administered so as to impair the general nutrition, lessens the biliary secretion; that given to dogs, in either small, gradually augmented, or in large doses, it does not increase the biliary secretion. He finds that it does not influence it at all so long as neither purgation nor impairment of health are produced.

As regards the other drugs employed, doses of podophylline, varying from two to eight grains, when given to dogs, diminished the solid constituents of the bile, whether they produced purgation or not. Doses which produced purgation lessened both the fluid and solid constituents. Doses of the solid extract of taraxacum, varying from 60 to 240 grains, affected neither the biliary secretion, the bowels, nor the general health of the animal.—*Practitioner, June, 1869, p. 355.*—*Braithwaite.*

